# A role for microsomal glutathione transferase 1 in melanin biosynthesis and melanoma progression

**DOI:** 10.1016/j.jbc.2023.104920

**Published:** 2023-06-14

**Authors:** Jie Zhang, Zhi-wei Ye, Lars Bräutigam, Paramita Chakraborty, Zhenwu Luo, John Culpepper, Muhammad Aslam, Leilei Zhang, Katarina Johansson, Jesper Z. Haeggström, Jianqiang Xu, Magnus Olsson, Danyelle M. Townsend, Shikhar Mehrotra, Ralf Morgenstern, Kenneth D. Tew

**Affiliations:** 1 Department of Cell and Molecular Pharmacology and Experimental Therapeutics, Medical University of South Carolina, Charleston, South Carolina, United States; 2 Department of Comparative Medicine, Karolinska Institutet, Stockholm, Sweden; 3 Department of Surgery, Medical University of South Carolina, Charleston, South Carolina, United States; 4 Department of Microbiology and Immunology, Medical University of South Carolina, Charleston, South Carolina, United States; 5 Pfizer Innovation AB, Sollentuna, Sweden; 6 Divisions of Biochemistry and Chemistry 2, Department of Medical Biochemistry and Biophysics, Karolinska Institutet, Stockholm, Sweden; 7 School of Life and Pharmaceutical Sciences (LPS) & Panjin Institute of Industrial Technology (PIIT), Dalian University of Technology, Panjin, China; 8 Division of Biochemical Toxicology, Institute of Environmental Medicine, Karolinska Institutet, Stockholm, Sweden; 9 Department of Drug Discovery and Biomedical Sciences, Medical University of South Carolina, Charleston, South Carolina, United States

**Keywords:** MGST1, melanin, melanoma, antioxidant capacity, oxidative stress, energy metabolism, tumor-infiltrating lymphocyte, survival

## Abstract

Recent advancements in the treatment of melanoma are encouraging, but there remains a need to identify additional therapeutic targets. We identify a role for microsomal glutathione transferase 1 (MGST1) in biosynthetic pathways for melanin and as a determinant of tumor progression. Knockdown (KD) of *MGST1* depleted midline-localized, pigmented melanocytes in zebrafish embryos, while in both mouse and human melanoma cells, loss of MGST1 resulted in a catalytically dependent, quantitative, and linear depigmentation, associated with diminished conversion of L-dopa to dopachrome (eumelanin precursor). Melanin, especially eumelanin, has antioxidant properties, and *MGST1* KD melanoma cells are under higher oxidative stress, with increased reactive oxygen species, decreased antioxidant capacities, reduced energy metabolism and ATP production, and lower proliferation rates in 3D culture. In mice, when compared to nontarget control, *Mgst1* KD B16 cells had less melanin, more active CD8^+^ T cell infiltration, slower growing tumors, and enhanced animal survival. Thus, MGST1 is an integral enzyme in melanin synthesis and its inhibition adversely influences tumor growth.

Abnormal accumulation of melanin is a characteristic trait of malignant melanomas, where historically, prognosis of patients with metastatic disease has been poor (median survival <1 year; 5-year mortality rate ∼90%) (1, 2). In the epidermis, melanin has important antioxidant properties and can act as a light filter, radioprotector, and scavenger of free radicals (3, 4, 5). Under normal physiological conditions, such properties are beneficial; however, in melanotic melanomas, melanin can imbue relative resistance to chemotherapy, radiotherapy, and phototherapy. Clinicopathological analysis on advanced melanomas has shown a negative correlation between tumor pigmentation and diseases outcome as defined by overall survival and disease-free time (6). Melanogenesis can generate highly reactive intermediates, which possess mutagenic and immunosuppressive activities, and it can stimulate glycolysis and hypoxia-inducible factor 1-alpha activation (7, 8), leading to melanoma progression. Inhibition of melanogenesis represents a plausible therapeutic approach for treatment of melanomas.

Mammalian melanocytes produce two distinct types of melanin in membrane-bound melanosomes, brown to black eumelanin, and yellow to reddish-brown pheomelanin (9). Eumelanin and pheomelanin derive from the common precursor dopaquinone, formed by the tyrosinase (TYR)-catalyzed oxidation of tyrosine to dopa and dopaquinone. In the presence of cysteine, dopaquinone transforms to cysteinyldopa, subsequently oxidized to cysteinyldopaquinone, which undergoes endocyclization to quinone imine, and rearranges to benzothiazine and eventually to pheomelanin. In addition to pheomelanin, dopaquinone can undergo slow intramolecular cyclization to cyclodopa, followed by rapid oxidation to dopachrome. Subsequently two additional TYR-related proteins (TYRP2 and TYRP1) catalyze the conversion of dopachrome to 5,6-dihydroxyindole-2-carboxylic acid and oxidation of 5,6-dihydroxyindole and 5,6-dihydroxyindole-2-carboxylic acid. Oxidation and polymerization of these indole moieties produce eumelanin.

Microsomal glutathione transferase 1 (MGST1) is a membrane-bound homotrimeric enzyme with three GSH-binding sites with a role in the biotransformation of lipophilic reactive electrophiles and reduction of membrane-embedded phospholipid hydroperoxides, displaying both GST and GSH peroxidase activities (10). Each activity is based on stabilizing a GSH thiolate (GS^-^) anion in the same active site, where arginine 130 plays an essential role (11). MGST1 displays broad subcellular distribution. In addition to endoplasmic reticulum and mitochondrial membrane (12), the enzyme has been detected in other organelle membranes including peroxisomes (13, 14) and the plasma (15, 16). While there are no published links between MGST1 and melanogenesis, MGST1 is abundant in retinal pigment epithelium (17), where cells have ample melanin granules (melanosomes) and form a dark enclosure for the photoreceptor system. MGST1 is transcriptionally increased under oxidative stress (18), which enhances antioxidant capacity in zebra finches, leading to the development of darker plumage coloration (19).

Like many cancers, melanoma cells have increased levels of reactive oxygen species (ROS). The balance between the antioxidant properties of melanin and generated ROS creates an environment unique to melanomas where altered redox flux and homeostasis can modulate signaling events at the ER-mitochondria interface, two compartments where MGST1 locates. A recent study showed MGST1 expression to be a prognostic risk factor for poor survival for melanoma patients (20). MGST1 is highly expressed in dedifferentiated and therapeutic-resistant human melanomas, and elevated levels are correlated with reduced overall survival of melanoma patients (data from Gene Expression Omnibus under accession number GSE80829 and The Cancer Genome Atlas Program database). The data supporting a role for MGST1 in both melanin biosynthesis and oxidative stress management led us to consider its role in melanoma disease progression.

## Results

### MGST1 regulates melanogenesis

**Figure 1:**
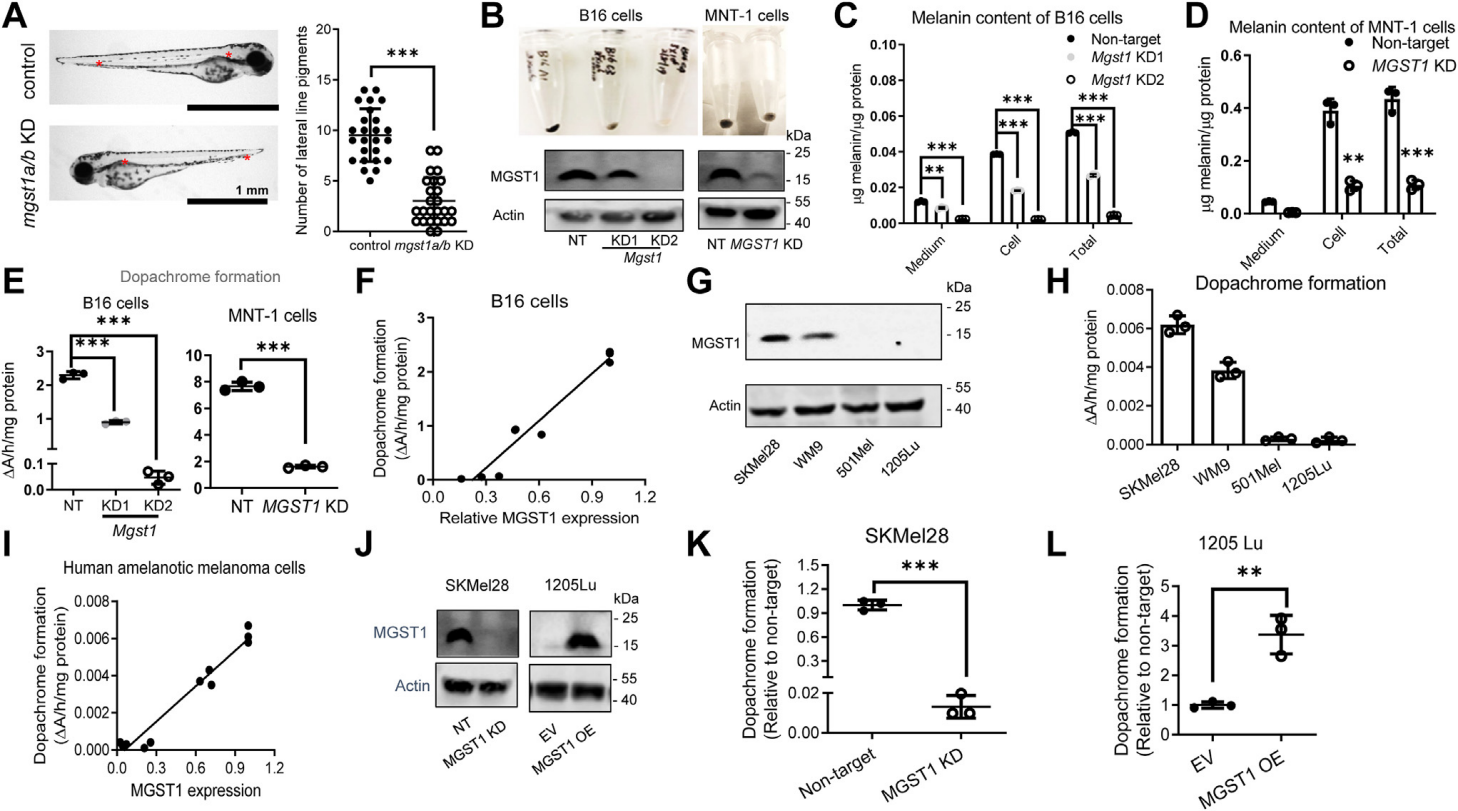
**MGST1 positively regulates melanogenesis.***A*, knockdown (KD) of *mgst1a/b* in zebrafish was achieved using antisense morpholinos. m*gst1a/b* KD zebrafish have decreased numbers of melanocytes localized in the midline of the embryos (between the two stars) compared to control morpholino zebrafish (n = 25/group). *B*, KD of *MGST1* in mouse B16 and human MNT-1 melanoma cells was achieved using lentiviral shRNA. MGST1 silencing was confirmed by Western blot. *MGST1* KD B16 and MNT-1 cell pellets show decreased pigmentation compared to nontarget (NT) shRNA control B16 and MNT-1 cell pellets. *C* and *D*, total melanin content (both intracellular and secreted) was measured by spectrophotometric assays in B16-NT, B16-KD1, B16-KD2, MNT-1 NT, and MNT-1 KD cells. *E*, dopachrome formation from L-dopa was measured in whole cell lysates from B16 NT, B16 *Mgst1* KD1, B16 *Mgst1* KD2, MNT-1 NT, and MNT-1 *MGST1* KD cells. *F*, linear correlation between dopachrome formation and amounts of MGST1 in B16 cells. *G*, MGST1 protein expression in amelanotic SK-Mel-28, WM9, 501Mel, and 1205Lu human melanoma cells. *H*, dopachrome formation from L-dopa was measured in whole cell lysates from SK-Mel-28, WM9, 501Mel, and 1205Lu cells. *I*, linear correlation of dopachrome formation with amounts of MGST1 in SK-Mel-28, WM9, 501Mel, and 1205Lu cells. *J*, KD or overexpression (OE) of *MGST1* in SK-Mel-28 cells or 1205Lu cells was achieved using lentiviral systems. MGST1 silencing or overexpression was confirmed by Western blot. *K* and *L*, dopachrome formation from L-dopa was measured in whole cell lysates from SK-Mel-28-NT, SK-Mel-28-KD (K), 1205Lu-EV, and 1205-OE (L) cells. ∗∗ and ∗∗∗ indicate *p* < 0.005, and *p* < 0.0005, respectively. MGST, microsomal glutathione transferase.

We previously designed antisense morpholinos to block the translational start site of the two *mgst1* isoforms in zebrafish. Depletion of *mgst1a/b* was confirmed by whole-mount immunohistochemistry (21), and the numbers of pigmented melanocytes localized in the midline of these zebrafish embryos were found to be significantly reduced (Fig. 1*A*, between the two stars). To extend the zebrafish data, we have generated pLKO.5 or pLKO.1 shRNA (*MGST1* or nontargeting (NT) control) lentiviral particles and showed that *MGST1* KD mouse B16 and human MNT-1 cells each exhibited a conspicuous depigmentation phenotype. Cell pellets, which were initially black, turned either gray or white (Fig. 1*B*). These data suggest a decrease in melanin production. In fact, less melanin pigment, both inside the cells and secreted into the medium, was found in *MGST1* KD B16 and MNT-1 cells (Fig. 1, *C* and *D*). Specifically, KD of *MGST1* in B16 or MNT-1 cells significantly reduced the conversion of L-dopa to dopachrome (Fig. 1*E*), the precursor step for the indo moieties of the eumelanic polymer, with a linear relationship between MGST1 expression and dopachrome formation (Fig. 1*F*).

Melanomas can be either melanotic or amelanotic. Of four amelanotic human melanoma cell lines, SK-Mel-28 and WM9 expressed high levels of MGST1, whereas no MGST1 expression was detected in 501Mel and WM9 (Fig. 1*G*). Although limited dopachrome formation was measured, amounts were linearly correlated with MGST1 levels (Fig. 1, *H* and *I*). We knocked down *MGST1* in SK-Mel-28 cells with pLKO.1 shRNA lentiviral particles and overexpressed *MGST1* in 1205Lu cells with pCDH MGST1 lentiviral particles and showed that in amelanotic melanoma cells, dopachrome formation correlated with the amounts of MGST1 (Fig. 1, *J*–*L*).

**Figure 2:**
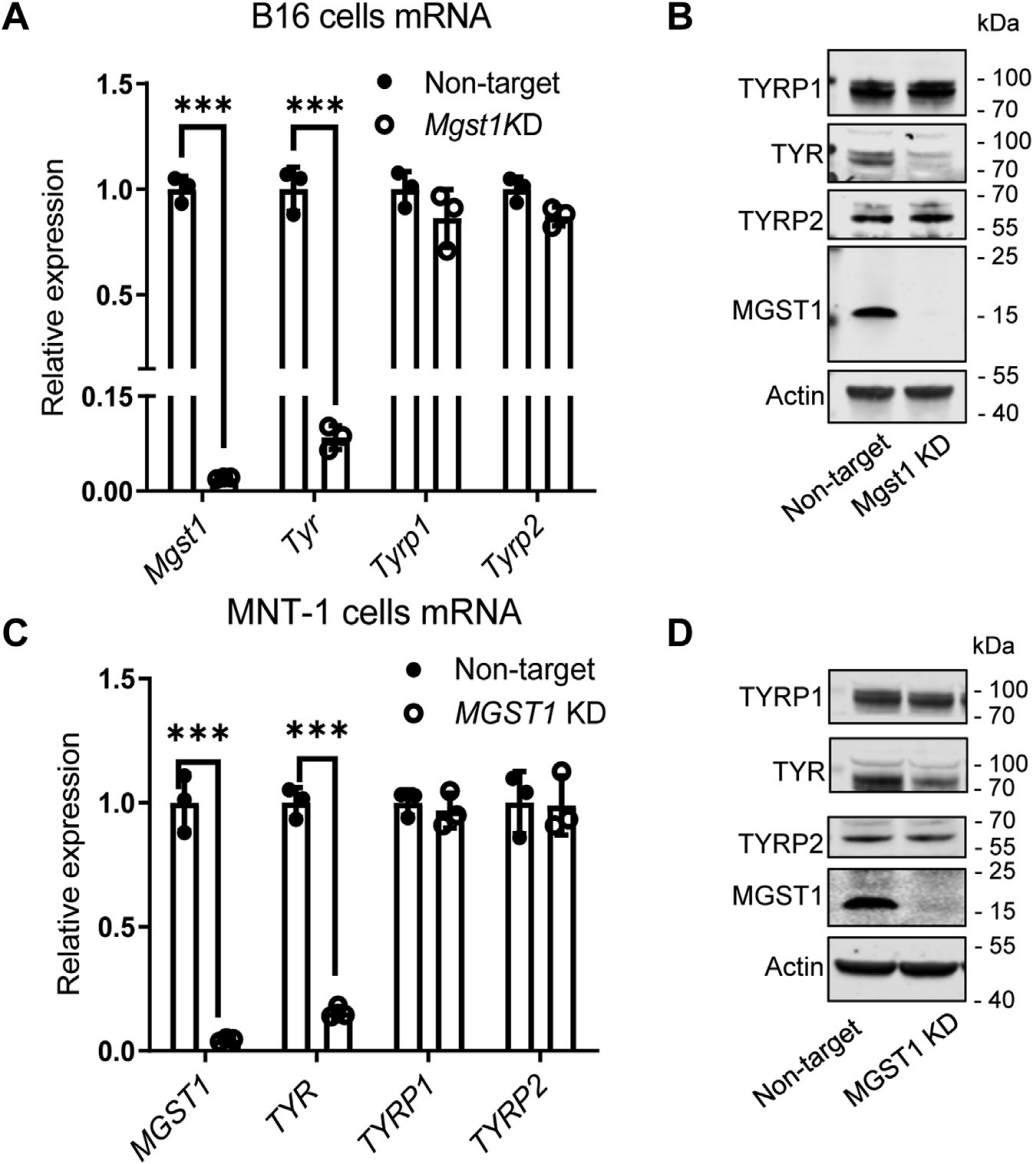
**Lack of MGST1 interrupts expression of tyrosinase, but not two other melanogenesis-associated enzymes, TYR-related protein 1 and 2.***A* and *B*, mRNA and protein expression of Tyr, Tyrp1, and Tyrp2 in B16 NT, B16 *Mgst1* KD cells. *C* and *D*, mRNA and protein expression of Tyr, Tyrp1, and Tyrp2 in MNT-1 NT and MNT-1 *MGST1* KD cells. ∗∗∗ indicates *p* < 0.0005. KD, knockdown; MGST, microsomal glutathione transferase; NT, nontargeting; TYR, tyrosinase; TYRP, tyrosinase-related protein.

Expression levels of TYR, TYRP1, or TYRP2, each of which is important for eumelanin synthesis, were examined. Both mRNA and protein levels of TYR were significantly lower in *MGST1* KD B16 (Fig. 2, *A* and *B*) and MNT-1 (Fig. 2, *C* and *D*) cells. No differences were observed in the expression of TYRP1 or TYRP2 in *MGST1* depleted cells (Fig. 2, *A*–*D*).

**Figure 3:**
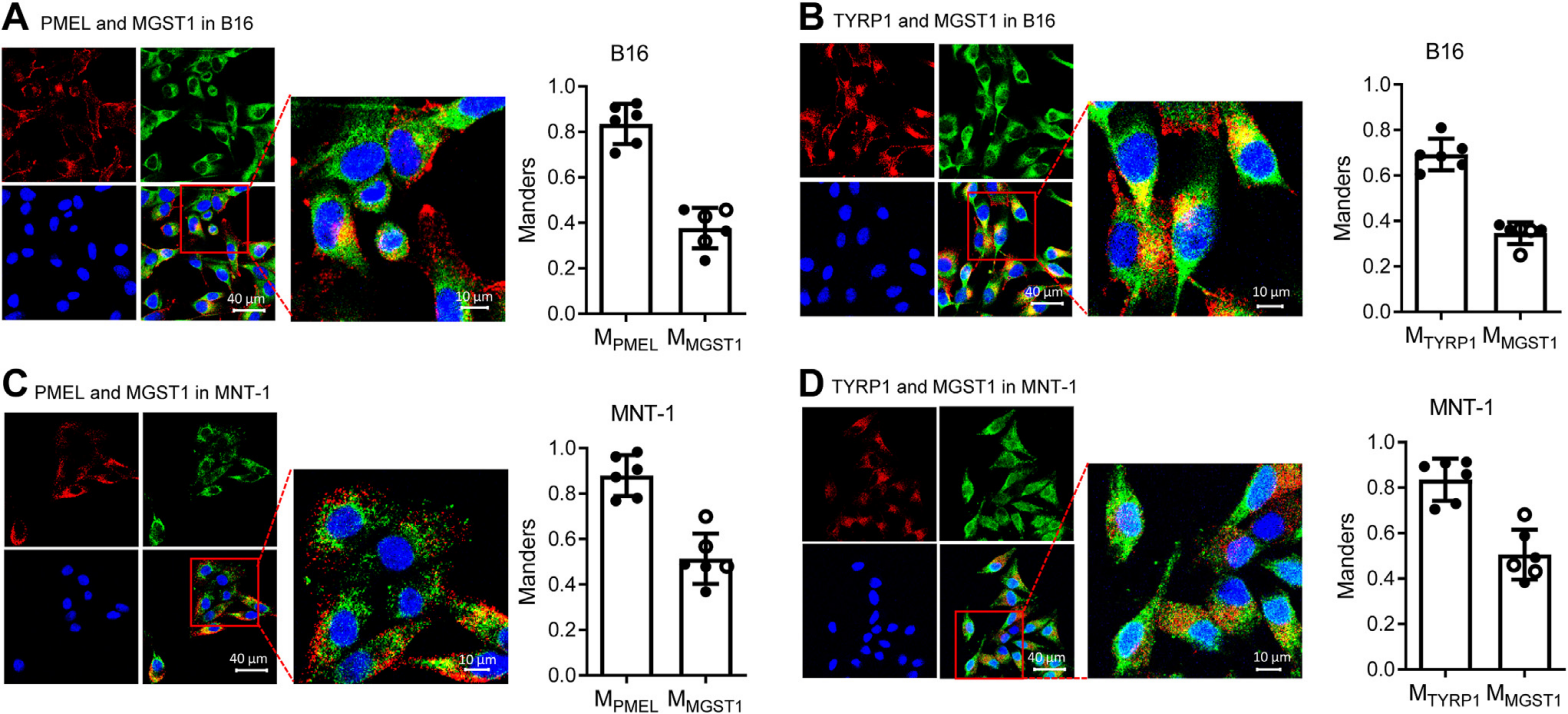
**MGST1 is spatially linked to melanosome markers PMEL and TYRP1.***A* and *B*, mouse B16 and (*C* and *D*) human MNT-1 cells were fixed and stained with anti-rabbit MGST1 and anti-mouse PMEL or anti-mouse TYRP1 antibodies along with Alexa-488 (*green* for rabbit) and Alexa-555 (*red* for mouse). *Green* and *red* fluorescence was imaged with an inverted Zeiss LSM880 laser scanning confocal microscope using a 40× water immersion super apochromat objective. Images shown are representative of three or more experiments. Colocalization was analyzed by Zen *Blue* software. *A* and *C*, *red* channel for anti-mouse PMEL and *green* channel for anti-rabbit MGST1. *B and D*, *red* channel for anti-mouse TYRP1 and *green* channel for anti-rabbit MGST1. Bar graphs showed the Manders coefficients for each channel. MGST, microsomal glutathione transferase; TYRP, tyrosinase-related protein.

To assess the localization of MGST1 in melanosomes, mouse B16 (Fig. 3, *A* and *B*) and human MNT-1 (Fig. 3, *C* and *D*) melanoma cells were immunofluorescently stained by using antibodies against MGST1 and melanosome-specific proteins PMEL and TYRP1. We quantified the colocalization of MGST1 with endogenous melanosome markers using the Mander’s overlap coefficient (M) (22), a value of 1 represents perfect correlation, M_MGST1_ is defined as the ratio of the "summed intensities of pixels from the green MGST1 image for which the intensity in the red PMEL/TYRP1 channel is above zero" to the "total intensity in the green MGST1 channel", and M_PMEL_/M_TYRP1_ is defined conversely. M_PMEL_/M_MGST1_ and M_TYRP1_/M_MGST1_ in B16 cells were 0.84/0.41 and 0.67/0.37. Similar results were found in MNT-1 cells; M_PMEL_/M_MGST1_ and M_TYRP1_/M_MGST1_ were 0.88/0.51 and 0.83/0.51, respectively. These results indicate that MGST1 is at least partially localized in melanosome membranes.

**Figure 4:**
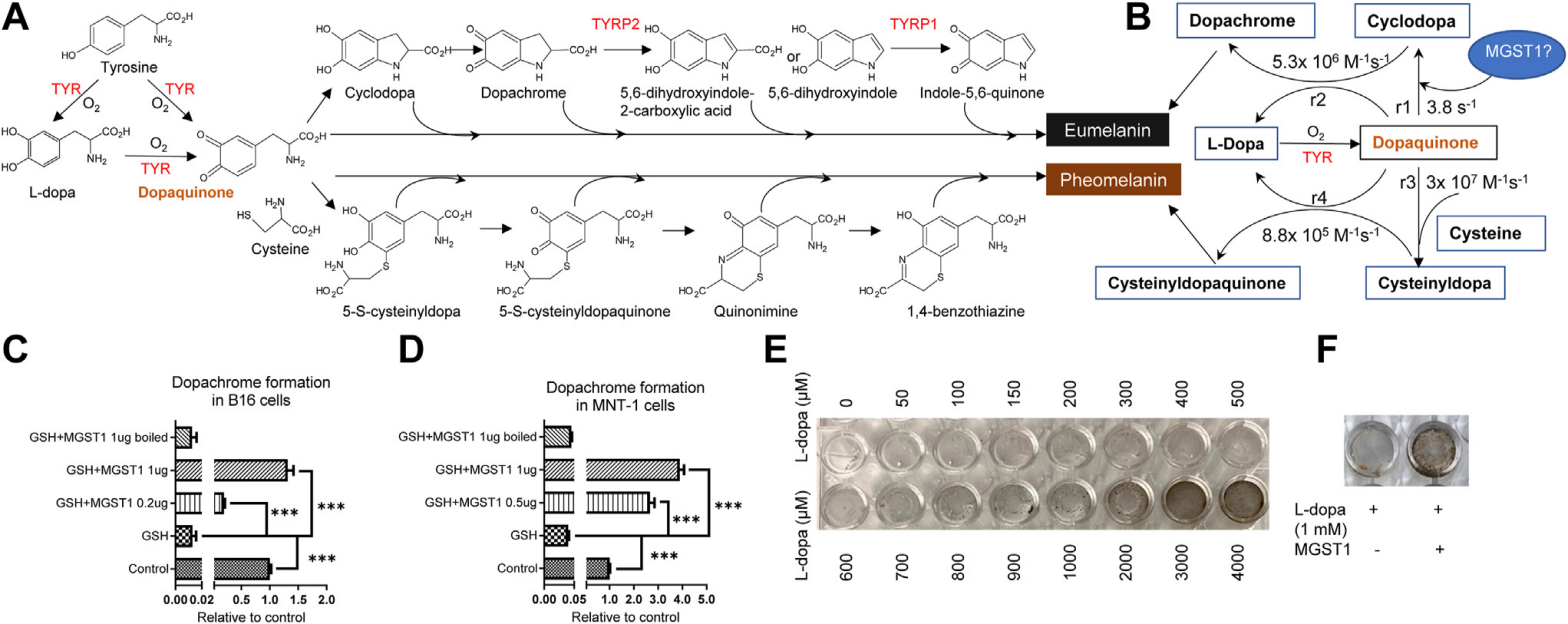
**MGST1, as a dopaquinone cyclase, promotes eumelanin formation.***A*, biosynthetic pathways leading to eumelanin and pheomelanin production. *B*, rate constants for the early-stage reactions. *C* and *D*, dopachrome formation from L-dopa was measured in whole cell lysates from B16 and MNT-1 cells in the presence of 5 mM GSH and recombinant MGST1 (active or inactivated). *E* and *F*, oxidation of L-dopa in the absence or presence of recombinant MGST1. ∗∗∗ indicates *p* < 0.0005. MGST, microsomal glutathione transferase; TYR, tyrosinase; TYRP, tyrosinase-related protein.

Eumelanogenesis and pheomelanogenesis diverge where dopaquinone undergoes either endocyclization to cyclodopa or addition of cysteine to cysteinyldopa. Assuming no other competing reactions, the ratio of cysteinyldopaquinone to dopachrome generation (r3 × r4 [Cys]/r1 × r2 (23)) represents the comparative rates of pheomelanogenesis and eumelanogenesis (Fig. 4, *A* and *B*) and is a linear function of cysteine concentration, with a slope of ∼1.3 × 10^6^ M^−1^. As dopaquinone is not available, we used L-dopa as an initial substrate, and dopaquinone was formed either by TYR, in the presence of B16 or MNT-1 lysates (Fig. 4, *C* and *D*), or by L-dopa oxidation in the absence of cell lysates (Fig. 4, *E* and *F*). To determine whether MGST1 catalyzes dopachrome formation, we included 5 mM GSH in the incubation (Fig. 4, *C* and *D*), as in the presence of 5 mM GSH, in order for the cyclization pathway to compete, a rate enhancement of around 10ˆ3 is needed, clearly requiring enzyme catalysis. As expected, dopachrome formation was decreased in the presence of GSH due to the formation of glutathionyldopa. Intriguingly, MGST1 can compete with GSH to drive the reaction toward dopachrome. This positively correlates with the amount of enzyme, and the inactivated control proved that the catalytically active enzyme is required (Fig. 4, *C* and *D*). In separate experiments, overnight incubation of L-dopa was utilized and eumelanin formation was measured in the presence of purified MGST1 enzyme. Pheomelanin formation was limited as cysteine was excluded. Pigment was formed as L-dopa concentrations increased, and recombinant MGST1 dramatically increased the amount of eumelanin produced (Fig. 4, *E* and *F*).

### Genetic modulation of MGST1 expression causes redox and metabolic changes in melanoma cells

**Figure 5:**
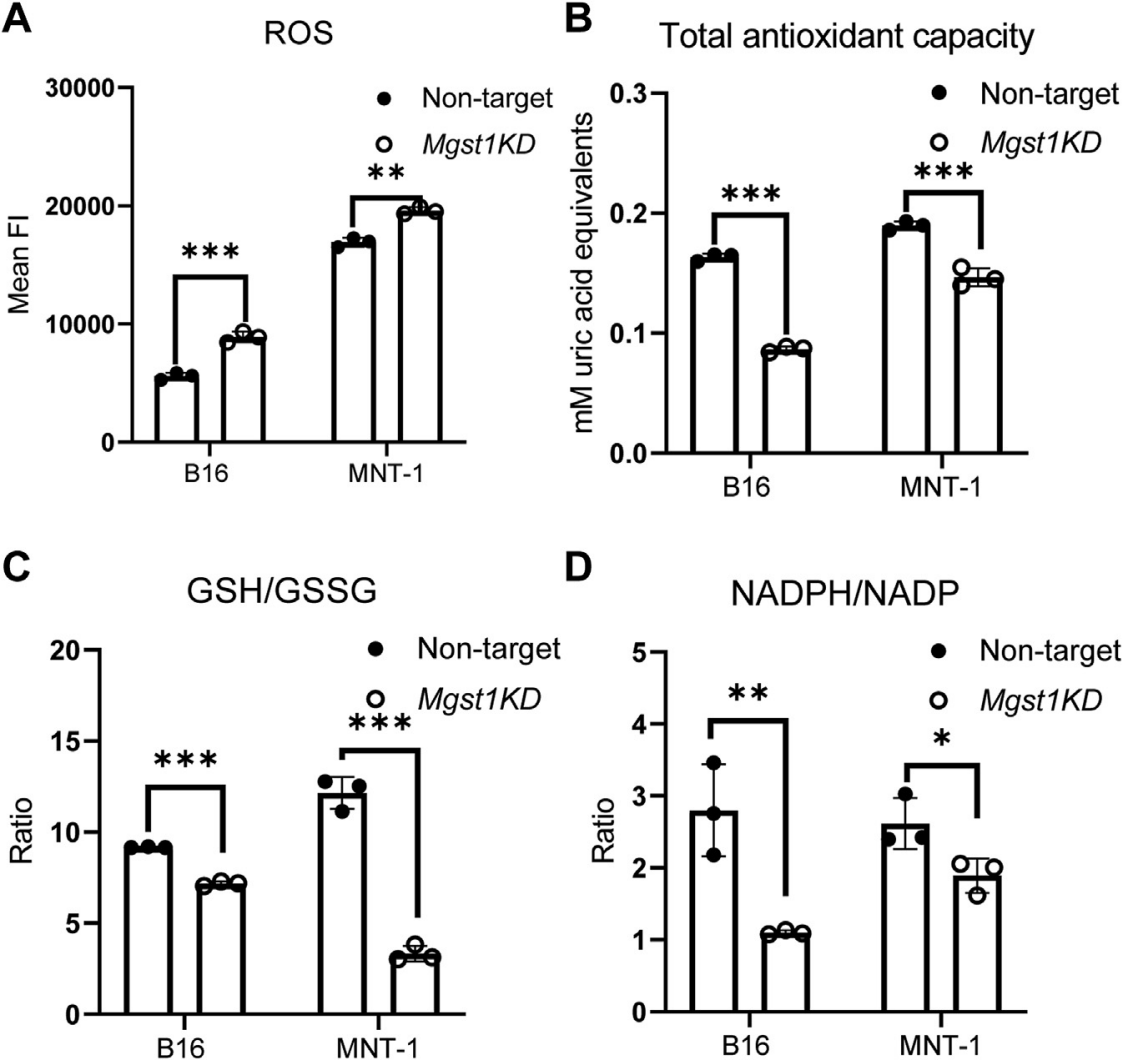
***MGST1* knockdown melanoma cells exhibit higher oxidative stress and lower antioxidant capacities.***MGST1* KD B16 and MNT-1 cells possess increased levels of ROS (*A*) and decreased total antioxidant capacities (*B*), with diminished GSH/GSSG (*C*) and NADPH/NADP (*D*) ratios. ∗, ∗∗, and ∗∗∗ indicate *p* < 0.05, *p* < 0.005, and *p* < 0.0005, respectively. KD, knockdown; MGST, microsomal glutathione transferase; ROS, reactive oxygen species.

MGST1 and eumelanin contribute to cellular redox homeostasis, thus KD of *MGST1* should influence their antioxidant capacities. In this regard, ROS, total antioxidant capacities, GSH/GSSG (oxidized GSH), and NADPH/NADP ratios were measured, and as hypothesized, parameters of oxidative stress were higher in both *MGST1* KD B16 and MNT-1 cells, with increased ROS levels and decreased total antioxidant capacities, GSH/GSSG, and NADPH/NADP ratios (Fig. 5, *A*–*D*).

**Figure 6:**
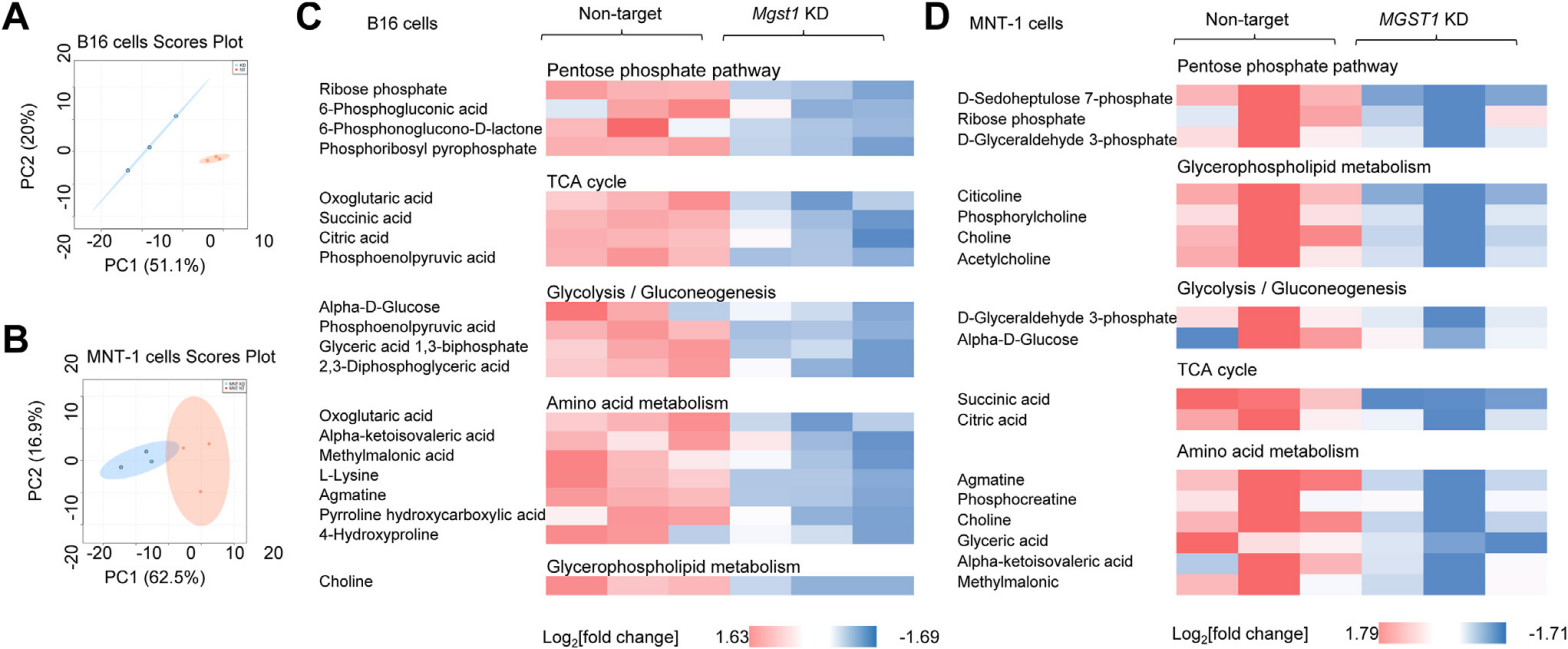
**Metabolic profiling of B16 and MNT-1 melanoma cells.** NT and *MGST1* KD cells were used for quantifying intracellular metabolites using LCMS. *A* and *B*, principal component analyses show distribution of the metabolites between NT and *Mgst1* KD B16 and MNT-1 cells. *C* and *D*, relative amounts of metabolites evaluated between NT and *MGST1* KD B16 (*C*) and MNT-1 (*D*) cells. KD, knockdown; MGST, microsomal glutathione transferase; NT, nontargeting.

**Figure 7:**
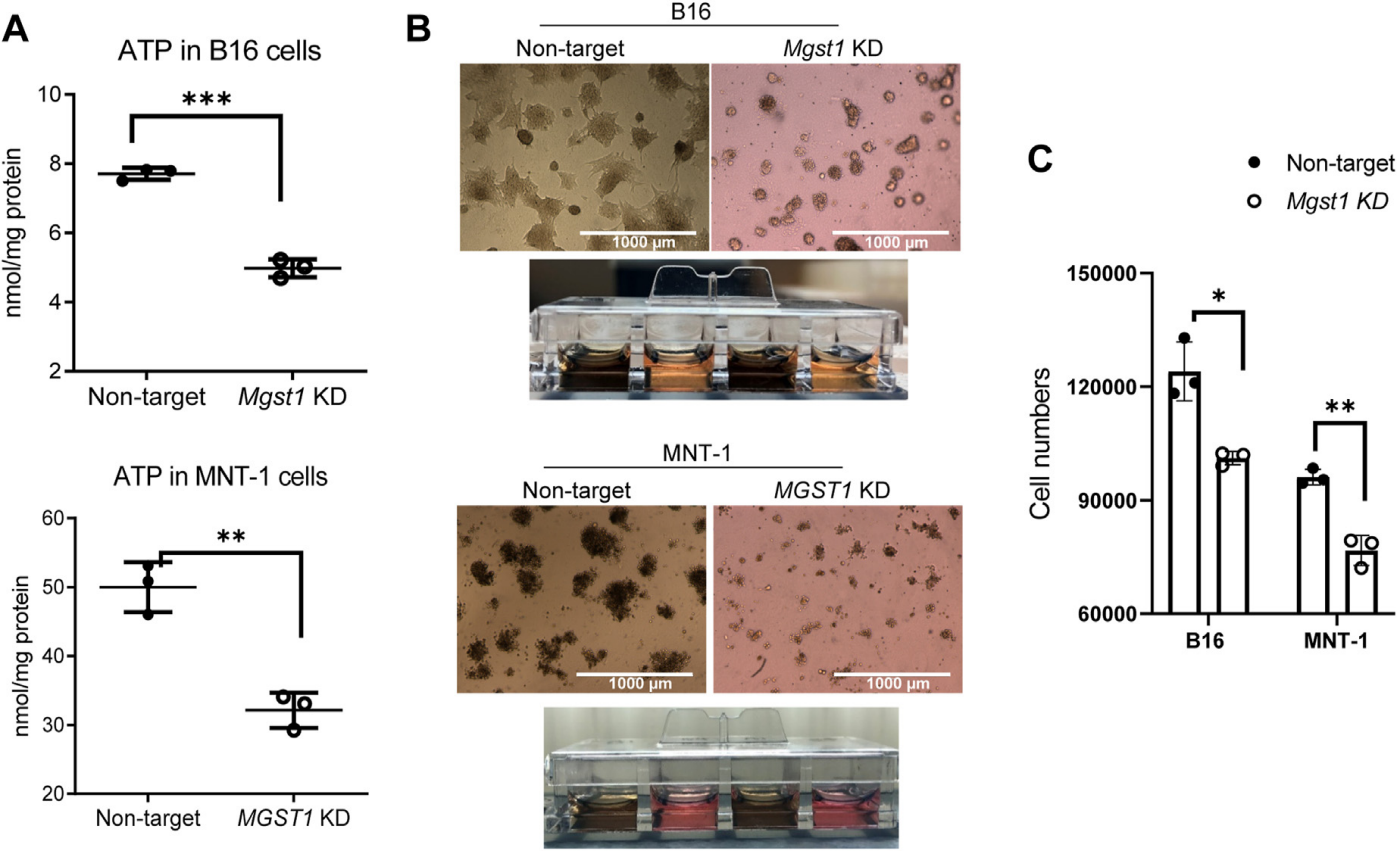
**Knockdown of *MGST1* suppresses melanoma migration *in vitro*.***A*, cellular ATP levels were measured where luminescence signals generated by luciferase are proportional to cellular ATP levels. *B*, after 10 days in melanoma cell medium, 3D-cultured B16 and MNT-1 KD cells showed significantly less morphological changes (dendrite formation). *C*, quantification of cell numbers was carried out by measuring DNA content using CyQuant dye. ∗, ∗∗, and ∗∗∗ indicate *p* < 0.05, *p* < 0.005, and *p* < 0.0005, respectively. KD, knockdown; MGST, microsomal glutathione transferase.

Melanomas are metabolically heterogeneous and able to use a variety of energy sources (24). We quantified the differences in metabolites accumulated within the *MGST1* KD *versus* NT cells. Principal component analyses in Figure 6, *A* and *B* summarize these differences. Figure 6, *C* and *D* show heat maps of key metabolic pathways comparing these metabolites. Specifically, the *MGST1* KD cells exhibited lower metabolites in the pentose phosphate pathway, tricarboxylic acid cycle, glycolysis, amino acid metabolism, and glycerophospholipid metabolism. These data indicate that *MGST1* KD produces distinct energy phenotypes, which have the capacity to impact tumorigenesis. Indeed, cellular ATP levels were lower in both *MGST1* KD B16 and MNT-1 cells (Fig. 7*A*). Morphological changes were observed in both B16 and MNT-1 *MGST1* KD cells, where KD cells were smaller and more condensed. After growth in melanoma cell culture medium for a few days, 3D-cultured B16 and MNT-1 KD cells showed significantly fewer signs of dendrite formation. The KD cells grew as single round flat acini devoid of protrusions, whereas the NT cells had a more transformed phenotype, growing as highly disorganized structures (Fig. 7*B*). Slower proliferation rates were observed in 3D-cultured KD cells (Fig. 7*C*).

### KD of *Mgst1* suppressed melanoma tumor progression and tumor-induced immunosuppression in C57BL/6J mice

**Figure 8:**
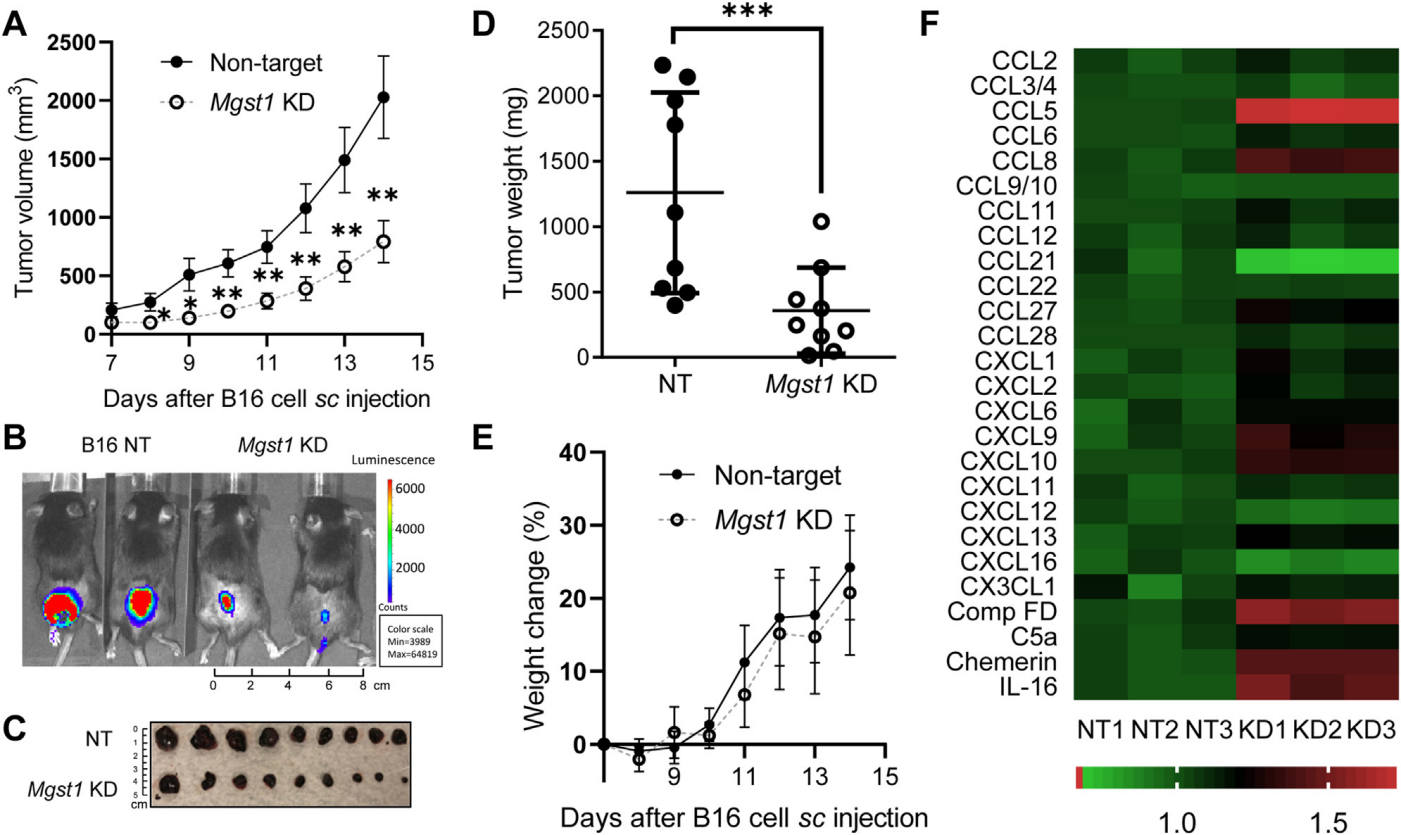
**Knockdown of *Mgst1* suppressed B16 melanoma tumor progression in C57BL/6 mice.** C57BL/6 mice were inoculated (subcutaneously, sc) with 0.3 × 10^6^ murine melanoma B16 NT and *Mgst1* KD cells on day 0, and starting on day 7, body weights and tumor sizes were measured every day. *A*, growth curves for B16 melanoma (n = 9/group). *B*, representative whole-body *in vivo* bioluminescent images of mice from each group at the end of study. *C*, digital photograph and (*D*) weights of excised B16 tumors at the end of study (n = 9/group). *E*, changes in the body weights of B16 tumor-bearing mice (n = 9/group). *F*, chemokine expression analyzed by R&D Chemokine Array. Tumor lysates were analyzed for protein content by bicinchoninic acid protein assay (n = 3/group). ∗, ∗∗, and ∗∗∗ indicate *p* < 0.05, *p* < 0.005, and *p* < 0.0005, respectively. KD, knockdown; MGST, microsomal glutathione transferase; NT, nontargeting.

B16 melanoma cells were subcutaneously implanted in the rear dorsal area of C57BL/6J mice. *In vivo*, *Mgst1* KD B16 cells showed a retarded growth rate compared to NT control cells. Solid B16 tumors formed and grew aggressively; however, the *Mgst1* KD tumors in mice were significantly smaller (Fig. 8*A*). B16 solid tumor growth was monitored by bioluminescence imaging (Fig. 8*B*). At the end of the study, mice were euthanized to collect tumor tissues (Fig. 8*C*). The average weights of tumors in mice inoculated with *Mgst1* KD B16 cells were significantly lower than in mice inoculated with NT control cells (Fig. 8*D*). The body weights of mice inoculated with *Mgst1* KD B16 cells were similar to the mice inoculated with NT control cells (Fig. 8*E*).

**Figure 9:**
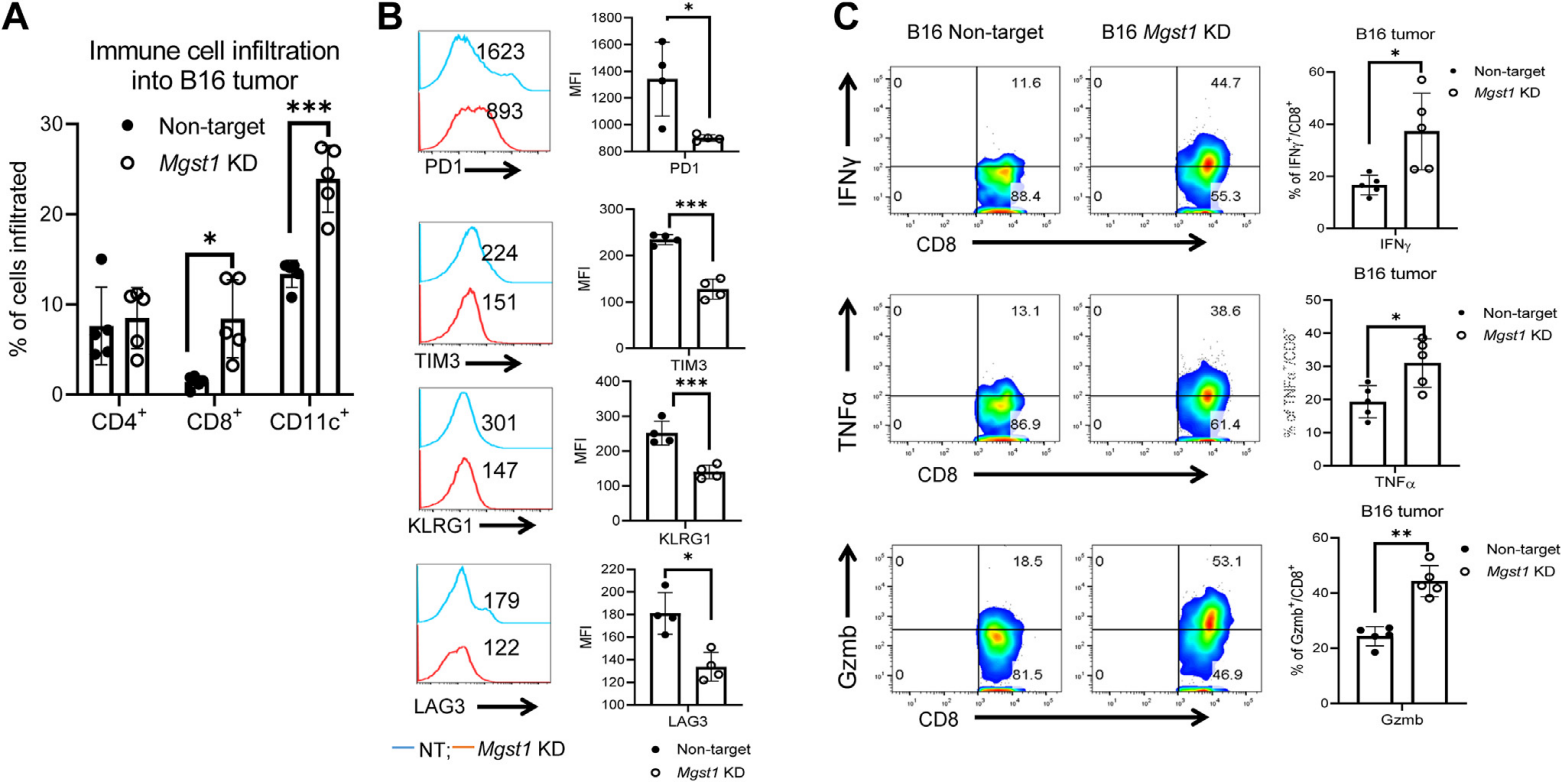
**Tumor-induced immunosuppression is reduced with the *Mgst1* KD strategy in C57BL/6 mice.***A*, frequency of intratumor lymphocytes analyzed by flow cytometry (n = 5/group). *B*, tumor-infiltrated lymphocytes retrieved from the tumor-bearing mice were used to determine either surface expression of PD1, TIM3, KLRG1, and LAG3 (n = 4/group) or (*C*) were activated *in vitro* with phorbol 12-myristate 13-acetate and ionomycin (500 ng/ml) and assessed for intracellular IFNγ, TNFα, and Granzyme B production using flow cytometry (n = 5/group). For (*B*), the adjacent bar graphs represent the cumulative data of mean fluorescence intensity of the cell surface expression of PD1, TIM3, KLRG1, and LAG3 on the tumor-infiltrated CD8^+^ T cells. For (*C*), the adjacent bar graphs represent the cumulative data of frequency of cytokine-secreting cells. ∗, ∗∗, and ∗∗∗ indicate *p* < 0.05, *p* < 0.005, and *p* < 0.0005, respectively. KD, knockdown; MGST, microsomal glutathione transferase.

To determine whether *Mgst1* KD affected melanoma cell secretion of cytokines and chemokines, intratumoral expression of C-C and C-X-C motif chemokines was analyzed. Chemokine levels changed in B16 *Mgst1* KD tumors as compared to NT control tumors. CCL5, CCL8, CXCL9, CXCL10, Comp FD, chemerin, and IL16 were robustly increased in *Mgst1* KD tumors as compared to controls (Fig. 8*F*). Quantification of tumor-infiltrating lymphocytes showed higher recruitment of CD8^+^ T cells and dendritic cells (CD11c^+^) to the tumor sites (Fig. 9*A*). Of significance, the expression of coinhibitory receptors (PD1, KLRG1, LAG3, and TIM3) was considerably lower in CD8^+^ T cells retrieved from B16 *Mgst1* KD tumor (Fig. 9*B*), and there was an increase in cytokine response (IFNγ and TNFα) along with Granzyme B (Fig. 9*C*). Given that the immune component becomes dysfunctional in the presence of large tumors, these data indicate that tumor-induced immunosuppression is also diminished with this *Mgst1* KD strategy.

## Discussion

We have shown in a teleost fish and two mammalian models that MGST1, in addition to its documented role in detoxifying peroxidation products, plays an important role in melanin production. TYR is considered as a primary rate-limiting enzyme in the early stages of melanin biosynthesis (25), and our results showed a reasonable degree of linearity between depletion of MGST1, reduction of TYR expression, and depigmentation. This functionality appears to be conserved across species, since KD of *MGST1* diminished melanin pigmentation in zebrafish, mouse, and human melanoma cells, implicating MGST1 as a key contributor to melanin biosynthesis. We found no indication that MGST1 was part of any protein complex with TYR, indicating that regulation of enzyme activity was not caused by any direct chaperone function of MGST1, unlike heat shock chaperone gp96 (26). In addition, MGST1 acts as a catalytic participant in the generation of eumelanin. Our data show that dopachrome formation (eumelanogenesis) in melanoma cells is stimulated in the presence of recombinant MGST1 enzyme but only when the catalytic activity of the enzyme is maintained. A cyclization pathway proceeds even in the presence of 5 mM GSH, where a rate enhancement of around 10ˆ3 is needed, clearly requiring enzyme catalysis. These results indicate that MGST1 may act as a dopaquinone cyclase, stabilizing the deprotonated dopaquinone. We appreciate the potential limits of this approach and understand that MGST1 may be active at more than one step; however, further study is restrained by the lack of usable melanogenesis intermediates.

We demonstrated the ubiquity of membrane distribution of MGST1 by showing its localization in melanosome membranes. The localization of MGST1 in different membranes is of particular significance, as the reactive lipophilic substrates, which concentrate in the membranes, cannot redistribute freely.

*MGST1* KD melanoma cells are under higher oxidative stress, with increased ROS levels, decreased antioxidant capacities, GSH/GSSG, and NADPH/NADP ratios, due to GSH peroxidase activity as well as the melanogenesis activity. Melanin is considered as an efficient antioxidant that scavenges reactive free radicals. GSH ethyl ester (a cell-permeable derivative of GSH, 5 mM), N-acetyl cysteine (2 mM), and vitamin E (20 μM) could not block the effects of MGST1 depletion on melanin synthesis (data not shown), implicating MGST1’s regulation of melanin synthesis is direct but not an indirect consequence of its effects on ROS levels. Pathways that regulate redox homeostasis are involved in melanoma initiation and progression and contribute to metastatic phenotype and drug resistance (27, 28). Enhanced ROS production triggers the occurrence and progression of melanoma, while concurrently, antioxidant systems are amplified to preserve sublethal ROS levels and favor cell survival and drug tolerance. High oxidative stress levels suppress melanoma metastasis, whereas adaptive changes in antioxidants increase metastatic spread (29). Therefore, our approach to block MGST1, melanogenesis, and enhance ROS levels can be a strategy for therapy.

Metabolic reprogramming is a characteristic of many tumors, and melanoma has been shown to be subject to altered amino acid and lipid metabolism and energy production (24). Both glycolysis and OXPHOS play a role in melanoma progression and ATP production, and the metabolic plasticity likely confers a significant survival advantage on melanoma cells (30). Our comparative analysis of the impact of *MGST1* KD in melanoma cells suggested altered expression of redox, energy, and metabolic pathways. Depletion of MGST1 conferred a phenotype imbalance in redox homeostasis and less dependence upon glycolysis and OXPHOS than NT counterparts. These changes served to impact growth rate and tumor development and are consistent with our results that reduced MGST1/melanin levels attenuated ATP progression and melanoma growth and with earlier observations that melanoma progression is impaired by simultaneously inhibiting respiratory complex I and lactate generation (31).

Given that some immunotherapies have provided complete remissions in malignant melanoma (32), we also showed that *MGST1*-depleted dorsal melanoma had enhanced chemokine production, with concomitantly increased attraction of CD8^+^ T cell and dendritic cells and decreased tumor growth. The B16 *Mgst1* KD tumor becomes more immunogenic than B16 NT tumor, with significantly reduced expression of negative, costimulatory molecules (PD1, KLRG1, LAG3, and TIM3 correlating with T-cell exhaustion or dysfunction (33)) in CD8^+^ T cells retrieved from B16 *Mgst1* KD tumor; there was also an increase in cytokine response (IFNγ and TNFα) along with Granzyme B. Therefore, we conclude that tumor-induced immunosuppression is reduced with the *Mgst1* KD strategy.

In summary, our present data are consistent with the observation that increased expression of MGST1 represents a risk gene for the survival of melanoma patients, particularly in the context of low MGST1 expression levels in normal skin (20). While at present there are no commercially available small molecule inhibitors of MGST1, the results reported here should serve as a platform to develop such drugs. It does not seem unreasonable to predict that pharmacologic inhibition of MGST1 would have effective antitumor effects in melanoma. As MGST1 also has an essential role in hematopoiesis (21), translational drug treatments would require careful analysis of marrow toxicity and therapeutic index.

## Experimental procedures

### Mice

C57BL/6J mice were obtained from the Jackson Laboratory (Strain #000664) and maintained in pathogen-free facilities. All mice experiments were performed in compliance with a referenced protocol (2018-00638) approved by the Institutional Animal Care and Use Committees of the Medical University of South Carolina. C57BL/6J mice were bred and housed under standard housing conditions, and group housing was up to five mice per cage. For tumor experiments, equal numbers of age- and gender-matched (both male and female) mice were randomly assigned for the experiments, when they were between 6 and 8 weeks old. No influence of sex on the results of the studies was observed.

### Zebrafish

Zebrafish were housed in standard conditions, and embryos were staged according to external morphological features (34). The WT line AB was used for all experiments. The morpholinos targeting zebrafish *mgst1a* (5′-TGCACAACTTCTGCCATCTTAGTTT-3′) and *mgst1b* (5′-GTCGCTGTTCATCAGATCGGCCATT-3′) from GeneTools have been described before (21). The morpholinos were dissolved to a concentration of 3 mM in ddH_2_O and diluted 1/10 in injection buffer (9 μM spermine, 0.21 mM spermidine, and 0.3% phenol red in PBS). Single-cell eggs were injected with 1.5 nl using a FemtoJet microinjector (Eppendorf). The standard control morpholino (5′-CCTCTTACCTCAGTTACAATTTATA- 3′) from GeneTools was used as control. Two days post fertilization, zebrafish embryos were manually dechorionated and photographed in brightfield using a Leica M165FC stereomicroscope equipped with a Leica DFC3000G camera. Lateral line pigment cells were quantified manually. A minimum of three biological replicates with minimum 15 embryos analyzed in each replicate were performed.

### Cell lines

B16-F1 melanoma cells were obtained from American Type Culture Collection (CRL-6323) and maintained in Dulbecco's modified Eagle medium (DMEM; Corning) supplemented with 10% fetal bovine serum (FBS) (Atlanta Biologicals) and 1× penicillin-streptomycin (Corning). MNT-1 melanoma cells were a kind gift from Prof Michael S. Marks (Children’s Hospital of Philadelphia and University of Pennsylvania) and maintained in DMEM medium supplemented with 20% FBS, 10% AIM-V medium (Gibco), 1× nonessential amino (Corning), and 1× penicillin-streptomycin. SK-Mel-28, WM9, 501Mel, and 1205Lu melanoma cells were kind gifts from Prof Philip H. Howe (Medical University of South Carolina) and maintained in RPMI medium (Corning) supplemented with 10% FBS and 1x penicillin-streptomycin. All cells were cultured at 37 °C in a humid atmosphere with 5% CO_2_.

### Viral production and transduction

To generate *MGST1* KD cell lines, pLKO.5 or pLKO.1 *MGST1* shRNA (mouse, Clone ID: TRCN0000302881; human, Clone ID: TRCN0000158997 and TRCN0000297898) and nontarget shRNA plasmids (SHC216 and SHC016) were purchased from Sigma. To generate *MGST1*-overexpressing cell lines, the complementary DNA (cdNA) of human *MGST1* gene from MM1.s multiple myeloma cells were amplified by PCR (Primer: Forward: 5′-CCG GAA TTC GCC ACC ATG GTT GAC CTC ACC CAG GT-3'; Reverse: 5′-CGC GGA TCC TTA CAG GTA CAA TTT ACT TTT CAG C-3') and subcloned into eukaryotic expression vector pCDH-CMV-MCS-EF1-Puro from System Biosciences (CD510B-1). The recombinant plasmid pCDH-MGST1 was sequenced by Eurofins Genomics with CMV primer 5′-CGCAAATGGGCGGTAGGCGTG-3'. Lentiviral vectors were then produced in HEK-293T cells (ATCC; CRL-3216). HEK-293T cells were seeded in 6 cm cell culture plates in DMEM medium supplemented with 10% FBS. When the cells reached 50 to 80% confluence, either pLKO-MGST1 shRNA, nontarget shRNA, pCDH-MGST1 or pCDH empty vector plasmid (1 μg), together with psPAX2 (750 ng; Addgene; #12259) packaging and pMD2.G (250 ng; Addgene; #12260) envelope plasmids were transfected into the cells using X-tremeGENE 9 (Sigma, #6365787001) according to the manufacturer’s instructions. After 48 h and 72 h of transfection, the lentivirus containing medium were collected, combined, and cleared by centrifugation at 500*g* for 5 min at 4 °C. The concentration of lentiviral particle was determined using quantitative PCR Lentivirus Titration Kit from Applied Biological Materials (LV900). Subsequently, melanoma cells were infected with the lentiviral vectors to either knockdown or overexpress MGST1. Cells were cultured in 6-well plates and infected with the lentivirus at a multiplicity of infection of 10 in the presence of polybrene (8 μg/ml). After 3 days of infection, melanoma cells with stable MGST1 underexpression or overexpression were selected with puromycin (3 μg/ml). Single colonies were selected, and knockdown or overexpression of MGST1 was quantified by real-time PCR and Western Blot. In some experiments, B16-F1 cells were first transduced with luciferase-Neo (Addgene; #105621) lentivirus and then with *Mgst1* shRNA lentivirus.

### 3D culture in a 4-well chamber slide

To obtain the 3D culture of B16-F1 and MNT-1 cells, matrigel (Corning)-coated 4-well chamber slides were used. Briefly, B16-F1 (5000 cells) or MNT-1 (10,000 cells) in 1 ml completed medium with 2% matrigel were seeded to each well of 80 μl matrigel-precoated chamber slides. The slides were incubated in the CO_2_ incubator, and the medium was changed every 4 days. Cells were imaged between 10 and 15 days with the EVOS FL Cell Imaging System.

### Immunofluorescence

B16 or MNT-1 cells were fixed in 4% formaldehyde and immunofluorescence staining applied according to previous protocols (35). After fixation, cells were incubated with primary antibodies: anti-MGST1 (Ab131059, 1:100 dilution) from Abcam and PMEL (MA1-34759, 1:50 dilution) or TYRP1 (MA5-12293, 1:50 dilution) from Thermo Fisher Scientific at 4 °C overnight, and then a secondary antibody conjugated with Fluor 488 (A32731, 1:200 dilution) or 555 (A32727, 1:200 dilution) from Thermo Fisher Scientific was applied. No staining was detected when primary antibodies were omitted. Slides were imaged with an inverted Zeiss LSM880 laser scanning confocal microscope using a 40× water immersion super apochromat objective. Images shown are representative of three or more experiments. Colocalization was analyzed by Zen Blue software (https://www.zeiss.com/microscopy/en/products/software/zeiss-zen-lite.html).

### RNA isolation and real-time quantitative PCR

Total RNA was extracted using the Isolate II RNA Mini kit (Bioline; #52073), and cDNA was generated with the iScript cDNA synthesis kit (Bio-Rad; #1708891) according to the manufacturers' protocols. Subsequently, quantification of gene expression was performed using iTaq Universal SYBR Green Supermix (Bio-Rad; #1725121) with detection on a MyiQ real-time PCR System (Bio-Rad) (36). The primers used are listed in Table 1. Relative gene expression quantification was based on the comparative threshold cycle method (2^−ΔΔCt^) with normalization of the raw data to the included housekeeping gene (GAPDH).

**Table 1:** Primer list for real-time PCR

Gene	Forward primer (5′ to 3′)	Reverse primer (3′ to 5′)
Mouse *Gapdh*	CCCAGCAAGGACACTGAGCAA	AGGCCCCTCCTGTTATTATGG
Mouse *Tyr*	CAGGCTCCCATCTTCAGCAGAT	ATCCCTGTGAGTGGACTGGCAA
Mouse *Tyrp1*	AGCCACAGGATGTCACTCAGTG	GCAGGGTCATATTTTCCCGTGG
Mouse *Tyrp2*	GCAAGATTGCCTGTCTCTCCAG	CTTGAGAGTCCAGTGTTCCGTC
Mouse *Mgst1*	TGCGACCGCATTCCAGAGGATA	TCCACCTTCTCGTCAGTGCGAA
Human *GAPDH*	GTCTCCTCTGACTTCAACAGCG	ACCACCCTGTTGCTGTAGCCAA
Human *TYR*	GCACAGATGAGTACATGGGAGG	CTGATGGCTGTTGTACTCCTCC
Human *TYRP1*	TCTCAATGGCGAGTGGTCTGTG	CCTGTGGTTCAGGAAGACGTTG
Human *TYRP2*	CTCAGACCAACTTGGCTACAGC	CAACCAAAGCCACCAGTGTTCC
Human *MGST1*	GCCAATCCAGAAGACTGTGTAGC	AGGAGGCCAATTCCAAGAAATGG

### RNA-seq and data analysis

Total RNA was extracted from B16-F1 and MNT-1 cells and subjected to commercial RNA-seq analysis (Novogene) (37). Transcript abundance directly reflects gene expression levels. The expected number of Fragments Per Kilobase of transcript sequence per Million base pairs sequenced was used to estimate gene expression levels. Volcano diagrams were plotted according to the different expression of genes from *MGST1* KD and NT cells, with the threshold of differential gene expression as follows: |log2 (Foldchange)| > 1 and q-value < 0.005.

### Western blot analysis

Total soluble protein was quantitated by bicinchoninic acid protein assay (Thermo Fisher Scientific; #23225), and equal amounts of protein were electrophoretically separated by SDS-PAGE (Bio-Rad) and transferred onto low fluorescent polyvinylidene difluoride membranes (Millipore Sigma) by a Trans-Blot Turbo Transfer System (Bio-Rad) (38). Polyvinylidene difluoride membranes were then probed at 4 °C overnight with appropriate primary antibodies: anti-TYR (#35-6000, 1:200 dilution), anti-TYRP1 (PA5-81909, 1:1000 dilution), and anti-TYRP2 (PA5-72571, 1:500 dilution) from Thermo Fisher Scientific, anti-MGST1 (Ab129175, 1:1000 dilution) from Abcam, and anti-beta actin (A5441) from Sigma. Immunoblots were then developed with IR fluorescence IRDye secondary antibodies (#926-68070; #926-68071; #926-32210; #926-32211; 1:15,000 dilution) from LI-COR, imaged with a two-channel (red and green) Odyssey CLx near-IR fluorescence imaging system (LI-COR), and quantified with Image Studio 4.0 software (https://www.licor.com/bio/image-studio/) (LI-COR).

### Metabolomics

The LCMS-based metabolomics study was performed by the Metabolomics Core Facility of Northwestern University. Melanoma cells were washed with ice-cold 0.9% NaCl and overlaid with ultracold HPLC grade methanol/water (80/20, v/v). The plates were incubated at −80 °C for 20 min, after which cells were scraped and collected. The cell suspensions were then centrifuged at 16,000*g* for 15 min at 4 °C, and the supernatant was transferred to a new tube and evaporated to dryness with a nitrogen stream. The dried metabolites were then shipped overnight on dry ice (39). Metabolite analyses were carried out in MetaboAnalyst (https://www.metaboanalyst.ca/MetaboAnalyst/ModuleView.xhtml) 5.0. Peak intensities were normalized to total protein concentrations for each cell line. Missing and 0 values were replaced with half minimum positive values in the original data assumed to be the detection limit. For heat maps of the two groups, *t* tests with an FDR cut-off value of 0.05 were used to identify significant differences.

### Human MGST1 expression and purification

Human *MGST1* cDNA was cloned in pPICZA vector (Thermo Fisher Scientific; V19520) and expressed in *Pichia pastoris* KM71H expression system. Cells were induced with 0.6% MeOH and harvested after 48 h of expression at 27 °C, resuspended in 50 mM Tris–HCl pH 7.8, 100 mM KCl, and 10% glycerol, and homogenized using bead beater (BioSpec Products). The membrane fraction was solubilized with 1% Triton X-100 and 0.5% sodium deoxycholate at 4 °C. Solubilized protein was purified by nickel-nitrilotriacetic acid affinity chromatography (GE Healthcare). The column was washed with 25 mM Tris–HCl pH 7.8, 500 mM NaCl, 10% glycerol, 5 mM 2-mercaptoethanol, 0.05% n-dodecyl β-D-maltoside (DDM), 0.1 mM GSH, and 20 mM imidazole, followed by 40 mM imidazole. MGST1 protein was eluted with 300 mM imidazole in the same buffer and further purified by S-hexylglutathione (Abcam and GE Healthcare) affinity chromatography. The column was washed in 25 mM Tris–HCl pH 8.0, 500 mM NaCl, 10% glycerol, 5 mM 2-mercaptoethanol, 0.05% DDM, and 0.1 mM GSH, and the protein was eluted with the same buffer containing 30 mM probenecid but without NaCl. The eluate was concentrated using Amicon Ultra 30-kDa centrifugal filter device (MilliporeSigma) and purified by size-exclusion chromatography using Superdex 200 16/600 (GE Healthcare) column eluted with 25 mM Tris–HCl pH 8.0, 100 mM NaCl, 10% glycerol, 0.1 mM tris(2-carboxyethyl)phosphine, 0.03% DDM, and 0.1 mM GSH. Purified MGST1 was concentrated to 32 mg/ml by ultrafiltration and stored at −80 °C.

### Flow cytometry

Staining for surface markers was performed by incubating cells with antibody at 1:200 dilutions in fluorescence-activated cell sorting buffer (0.1% BSA in PBS) for 30 min at 4 °C. For intracellular cytokine staining of IFNγ and TNFα, surface markers were stained before fixation/permeabilization (BD Cytofix/Cytoperm Kit, #554714; BD Biosciences) (40). Flow antibodies were purchased from BioLegend with the information as follows: CD8a-BV786 (#563332), CD11c-BV786 (#563735), PD1-PE/Cy7 (#109109), TIM3-APC (#119705), KLRG1-FITC (#138409), LAG3-PerCP/Cy5.5 (#125211), TNFα-PE/Cy7 (#506323), and Granzyme B-Alexa 700 (#372221). Samples were acquired on BD LSRFortessa flow cytometer and analyzed with FlowJo software (https://www.flowjo.com/solutions/flowjo) (Tree Star) unless otherwise stated.

### Chemokine protein arrays

Chemokine protein levels in tumor cell supernatant (500 μl) or solid tumor lysates (200 μg) were determined by human or mouse chemokine array kit (R&D system; ARY017 and ARY020). Immunoblots were developed with IRDye 800CW streptavidin secondary antibodies (LI-COR; #926-32230, 1:15,000 dilution), imaged with Odyssey CLx near-IR fluorescence imaging system, and quantified with Image Studio 4.0 software.

### Measurement of intracellular protein thiol, GSH, and GSSG levels

Cell lysates were collected and immediately subjected to protein thiol and GSH measurement as reported (41, 42, 43). Fluorescent intensities were detected at 400Ex/465Em by CLARIOstar microplate reader (BMG LABTECH). Quantitative determinations of GSH and GSSG levels were performed using the enzymatic recycling method. Briefly, protein in the cell extracts was precipitated by sulfosalicylic acid, and the supernatant was then divided into two parts. For reduced GSH, the supernatant was incubated with thiol fluorescent probe IV (Sigma; #595504), and fluorescence intensities were measured. For total GSH (GSH + GSSG), the supernatant was neutralized by triethanolamine and incubated with the reduction system, containing NADPH (Sigma; #10107824001) and GSH reductase (Sigma; #10105678001), at 37 °C for 20 min. GSSG was calculated based on the results from reduced GSH and total GSH; the ratio of GSH/GSSG = [GSH]/(([Total GSH] − [GSH])/2).

### Measurement of intracellular NADPH and NADP levels

Cell lysates were collected and immediately subjected to NADPH and NADP measurement by the NADP/NADPH Assay kit (Abcam; ab176724) according to the manufacturer’s protocol. The results of fluorometric assays at 540Ex/590Em were obtained using a CLARIOstar microplate reader.

### ROS detection

Cells were incubated with 1 μM 2′,7′dichlorodihydrofluorescein diacetate (H_2_DCFDA, D399; Thermo Fisher Scientific) for 45 min, followed by two times wash with PBS. The fluorescent signals were detected by a Beckman CytoFLEX S flow cytometer (Beckman Coulter) and analyzed with CytExpert (https://www.beckman.com/flow-cytometry/research-flow-cytometers/cytoflex/software) 2.1 software (Beckman Coulter) in the Analytical Redox Biochemistry Core at Medical University of South Carolina (44).

### Total antioxidant capacity assay

Antioxidant capacity assays were performed by The OxiSelect Total Antioxidant Capacity Assay kit (Cell Biolabs; STA-360) according to the manufacturer’s protocol. The results of colorimetric assays at 490 nm were obtained using a CLARIOstar microplate reader.

### TYR activity assay based on dopachrome formation

L-Dopa was used as the starting material, and the formation of dopachrome, which has an absorbance maximum at 475 nm (45), measured spectrophotometrically with or without recombinant MGST1. In some cases, cell lysates and GSH were included. Whole cell pellets were lysed in 0.1 M potassium phosphate buffer pH7.0 containing 0.1% Triton X-100 and centrifuged at 16,000*g* for 10 min. The supernatant was used for the following experiments. After protein quantification with the bicinchoninic acid protein assay kit, equal amounts of protein (20∼150 μg) were aliquoted into 96-well plates. Enzyme reactions were initiated by adding 1 mM L-Dopa (Sigma; #37380), and the absorbance was measured.

### Total melanin determination

Melanin pellets from the cell-free culture supernatants of melanoma cells or cells (precipitated after cell lysate preparation) were washed with PBS and boiled in 1 M NaOH containing 10% dimethyl sulfoxide for 15 min. The absorbance of the dissolved melanin solution at 470 nm was measured. Standard curve was prepared with synthetic melanin (MP Biomedicals; #0215534380) at 7.81∼500 μg/ml.

### ATP determination

The cellular ATP levels were measured with a luminescence-based ATP Determination Kit (Thermo Fisher Scientific; A22066) according to the manufacturer’s protocol. The luminescence signals generated by luciferase are proportional to the cellular ATP levels.

### Cell proliferation assay

Quantification of cell numbers was carried out by measuring DNA content using CyQuant dye (Thermo Fisher Scientific; C7026). Fluorescence was measured with a CLARIOstar microplate reader at 485Ex/535Em. The standard curve was made with B16 and MNT-1 cells from 1000 to 32,000 cells per 100 μl sample.

### Isolation of TILs

To obtain tumor-infiltrating lymphocytes (TILs) from C57BL/6J mice bearing established subcutaneous B16 melanoma, solid tumors were excised, chopped finely using tweezers and scissors, and digested with 1 mg/ml collagenase D (Sigma; #11088866001) and 1000 IU/ml DNAse I (Sigma; #4716728001) for 60 min at 37 °C. Following digestion, the fragments were filtered through 40 μm cell strainers (Greiner Bio-One). After removal of red blood cells with ammonium-chloride-potassium lysis buffer (Gibco; A10492), the cells were resuspended in 4 ml RPMI medium and layered carefully over 4 ml Ficoll-paque (GE Healthcare; #17-1440-02), followed by centrifugation at 1025*g* for 20 min at room temperature. The enriched TILs, obtained at the interface as a thin buffy layer were washed with PBS and finally resuspended in FACS staining buffer for further staining procedures. For each mouse, lymphocytes from spleen were also harvested.

### Mouse model of subcutaneous melanoma

B16-F1 control or *Mgst1* KD melanoma cells (3 × 10^5^) were injected subcutaneously into dorsal skin of 6 to 8 weeks old C57BL/6J mice. Seven days after inoculation, body weights and tumor sizes were measured daily. Animals were sacrificed at day 14 after cancer cell inoculation and tumors were harvested.

### Bioluminescent imaging

Mice were imaged using an IVIS Imaging System 200 Series (Caliper Life Sciences) in conjunction with the PerkinElmer’s XGI-8 Gas Anesthesia System. Mice were injected intraperitoneally with 100 μl of PBS containing D-luciferin monopotassium salt (15 mg/ml; Bio-Vision, #7903) 5 min before imaging, followed by general anesthesia 2 min before imaging. The exposure time of images ranged from 1 to 10 min depending on signal intensity. The bioluminescence signal was quantified with “region of interest” measurement tools in Living Imaging software (https://www.perkinelmer.com/Product/li-software-for-lumina-1-seat-add-on-128110).

## Quantification and statistical analysis

Statistical analyses were performed with GraphPad Prism 8 (GraphPad) or Student’s two-tailed *t* tests. ∗, ∗∗, and ∗∗∗ indicate *p* < 0.05, *p* < 0.005, and *p* < 0.0005, respectively. Data were expressed as mean ± SD of three independent experiments. In animal experiments, n represents number of animals utilized in each treatment group.

## Data availability

All data supporting the findings of this study are available within this manuscript. Cell lines generated in this study are available upon request from Dr Jie Zhang (zhajie@musc.edu).

## Conflict of interest

The authors declare that they have no conflicts of interest with the contents of this article.
